# A Case of Human Epidermal Growth Factor Receptor 2‐Positive Colon Cancer With Invasive Micropapillary Carcinoma Component

**DOI:** 10.1002/deo2.70184

**Published:** 2025-08-14

**Authors:** Masashi Kono, Yoriaki Komeda, Hiroshi Kashida, Satoru Hagiwara, Akihiro Yoshida, Shunsuke Omoto, Mamoru Takenaka, Naoko Tsuji, George Tribonias, Masatoshi Kudo

**Affiliations:** ^1^ Department of Gastroenterology and Hepatology Faculty of Medicine Kindai University Osaka Japan; ^2^ Department of Gastroenterology Red Cross Hospital Athens Greece

**Keywords:** colon cancer | endoscopic submucosal dissection | endoscopic ultrasonography | IMPC | invasive micropapillary carcinoma

## Abstract

A female patient in her 60s tested positive for the fecal occult blood test while undergoing health screening. Colonoscopy revealed a 15‐mm‐sized flat elevated lesion with a central depression in the sigmoid colon. Narrow‐band imaging magnification revealed a Japan Narrow‐Band Imaging Expert Team classification of type 2B, whereas crystal violet staining showed a mild to severely irregular type VI pit pattern. Ultra‐magnification imaging revealed an EC3a morphology in the depressed area. Endoscopic ultrasonography revealed partial disruption of the third layer, leading to the diagnosis of T1b (SM) colon cancer. Owing to the intermediate lesion size and since the patient had requested it, an endoscopic submucosal dissection was performed as an initial treatment. Pathological analysis revealed a moderately differentiated tubular adenocarcinoma with an invasive micropapillary carcinoma (IMPC) component, with deep submucosal invasion. Additional surgery was performed, and no recurrence was observed in the following three years. IMPC is known for its high rate of lymph‐node metastasis and poor prognosis, as reported for breast, bladder, and lung cancers. IMPC is rare; this report presents a literature review and case details. This case represents the first reported instance of identification of a cancerous IMPC component by magnifying endoscopy at the T1b (SM) depth. Thus, even for intermediate lesions, IMPC should be considered as a differential diagnosis when endoscopic imaging suggests malignancy.

## 1 Introduction

Invasive micropapillary carcinoma (IMPC) was first described in 1993 by Siriaunkgul et al. [1] as a biologically aggressive subtype of invasive ductal carcinoma of the breast. It is characterized by a high frequency of lymphovascular invasion and lymph node metastasis [2].

Reports of IMPC in organs such as the urinary tract, lungs, and salivary glands have since emerged, and all provide evidence of associated poor prognosis due to frequent lymphatic spread. Herein, we report an unusual case of early‐stage colorectal carcinoma with an IMPC component.

## 2 Case Report

**FIGURE 1:**
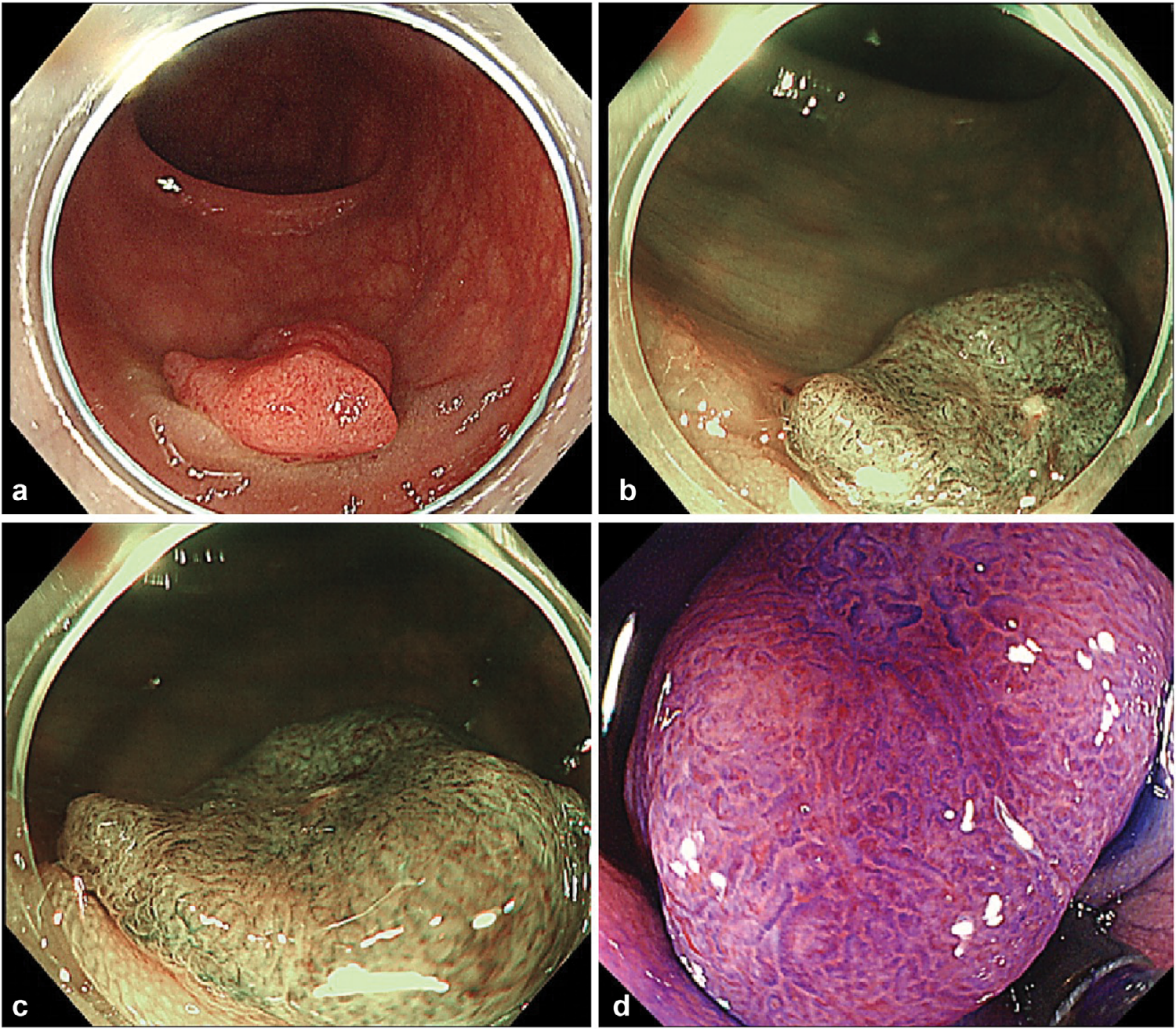
(a) Endoscopic images of white light imaging (WLI), (b) narrow band imaging (NBI) (normal), (c) NBI (magnification), and (d) crystal violet staining (magnification).

**FIGURE 2:**
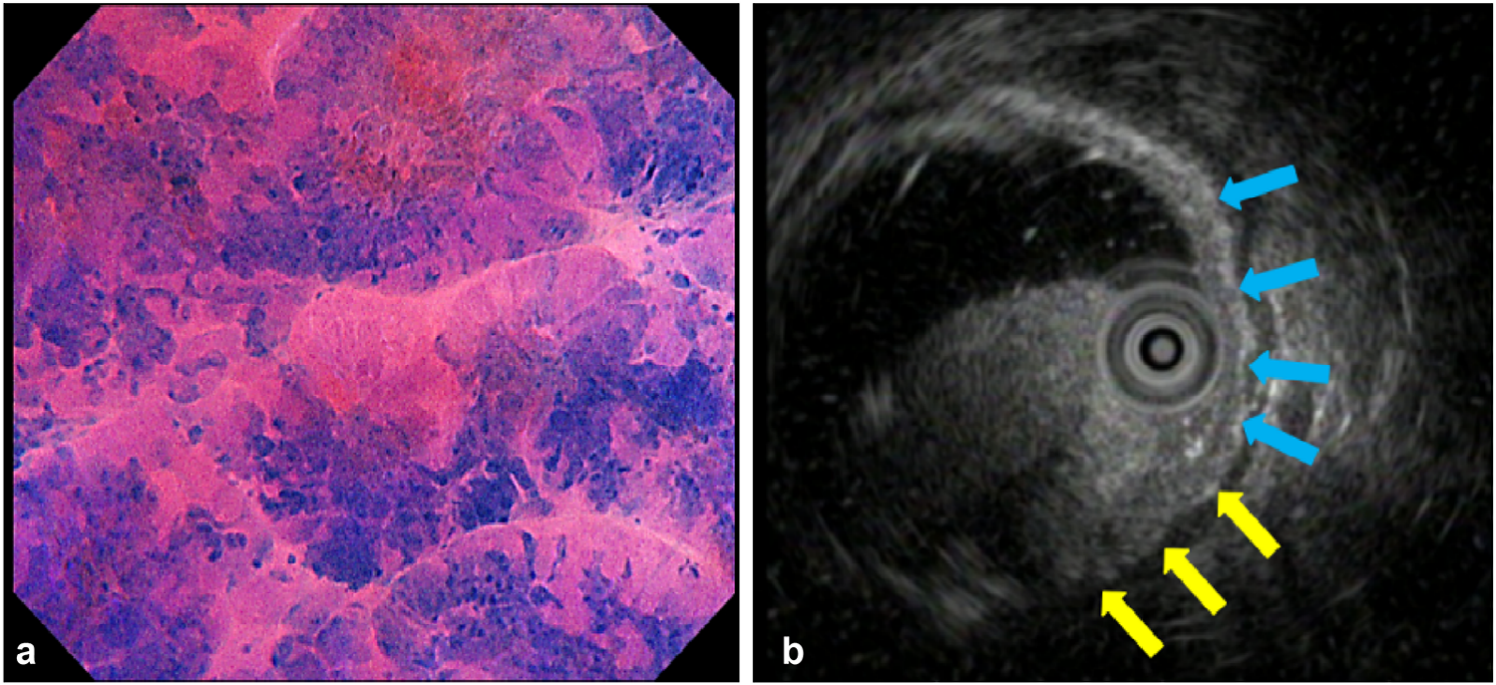
(a) An ultra‐magnified endoscopic image classified as EC3a; (b) An endoscopic ultrasonography image. The area indicated by the blue arrow can be traced in the third layer (submucosal layer), but it appears to be interrupted (tumor invasion) at the area marked by the yellow arrow.

A female patient in her 60s was referred for further evaluation after a positive fecal occult blood test. Colonoscopy identified a 15 mm, red, flat, elevated lesion in the sigmoid colon, surrounded by white plaques, but there were no obvious signs of pulling or distortion of the folds (Figure 1a). Magnified narrow‐band imaging revealed a Japan Narrow‐Band Imaging Expert Team classification of type 2B (Figure 1b,c), whereas crystal violet staining revealed a mild to severely irregular type VI pit pattern (Figure 1d). Ultra‐magnification imaging of the depressed center revealed preserved glandular structures but pronounced structural atypia, nuclear polarity disruption, and a high nucleus‐to‐cytoplasm ratio, classified as EC3a (Figure 2a).

**FIGURE 3:**
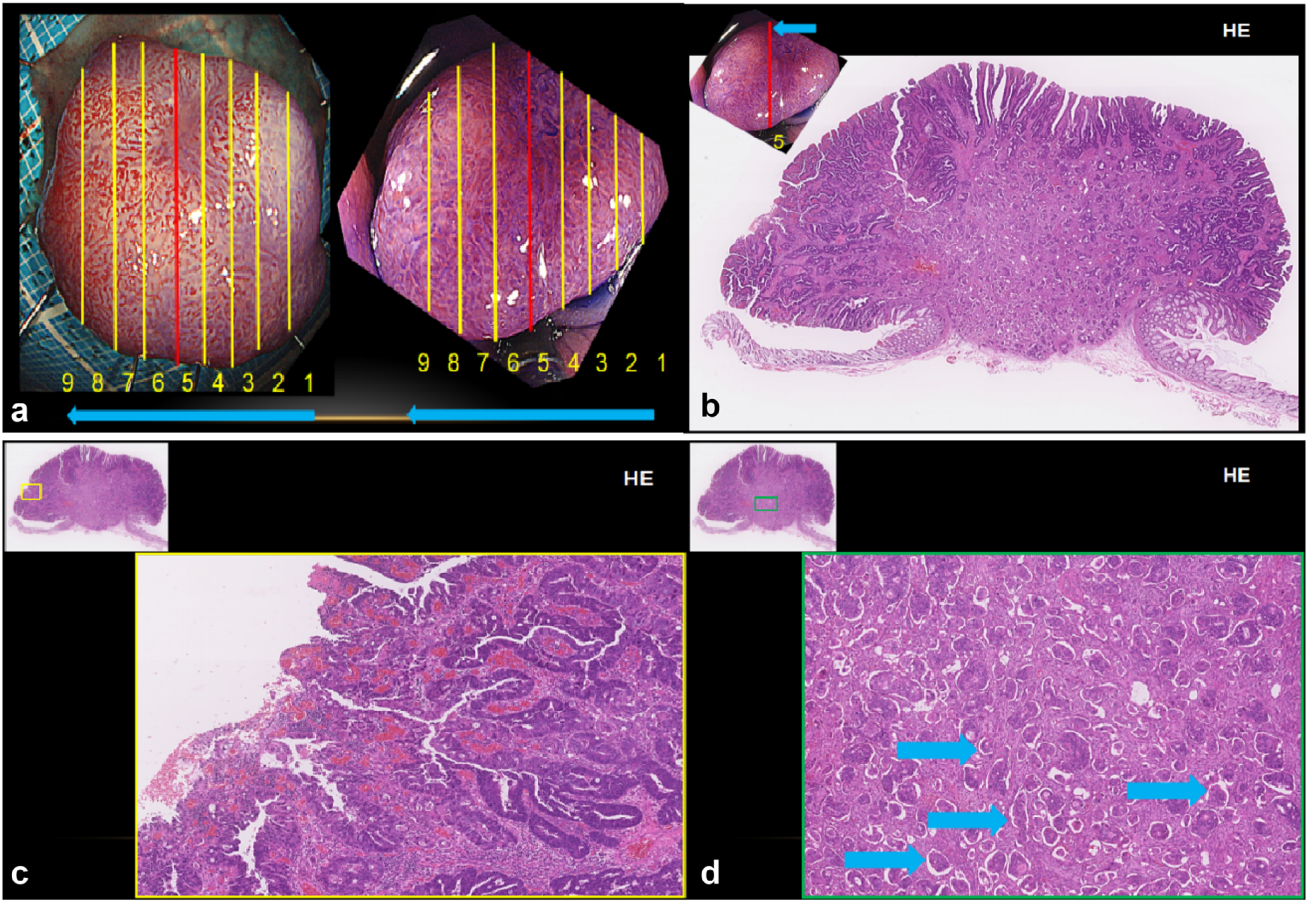
Pathological findings from hematoxylin‐eosin (HE) staining: (a) the sectioned specimen; (b) Section 5 macro image; (c) Section 5 weak magnification image. (d) Section 5 strong magnification image. The blue arrow indicates invasive micropapillary carcinoma (IMPC).

**FIGURE 4:**
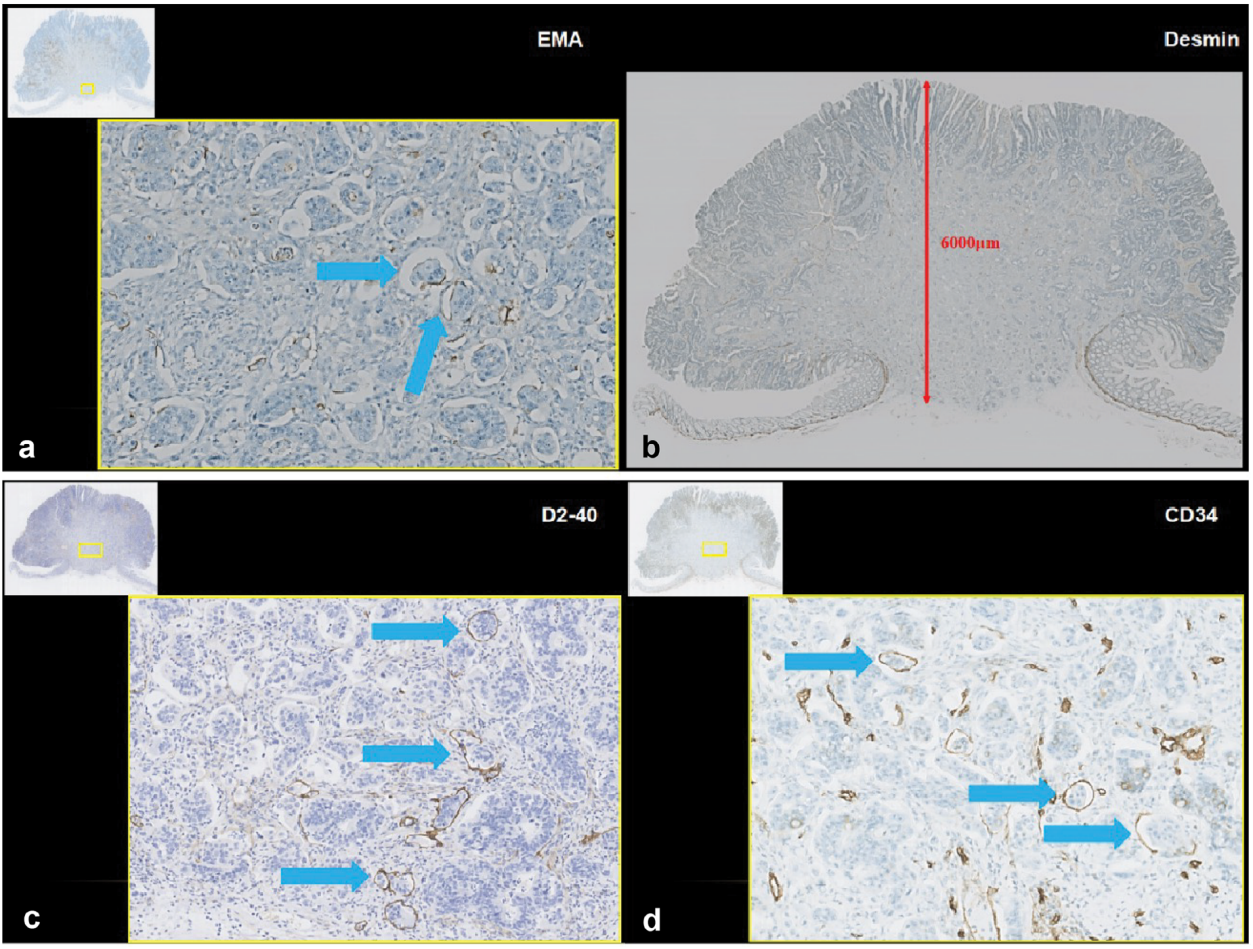
(a) Pathological findings from: epithelial membrane antigen (EMA) staining, (b) Desmin staining, (c) D2‐40 staining, and (d) CD34 staining. These immunohistochemical findings suggested invasive micropapillary carcinoma (IMPC) of the colon.

Endoscopic ultrasonography identified a discontinuity in the third layer, leading to a clinical diagnosis of T1b (SM) colon cancer (Figure 2b). Owing to the intermediate size of the lesion and the patient's preference, endoscopic submucosal dissection (ESD) was performed. Pathological findings described moderately differentiated tubular adenocarcinoma with a micropapillary component (0‐Is, 15×12 mm, tub2, pT1b [SM, 6000 µm], IFN‐b, Ly1b [D2‐40], V1b [CD34], BD2, HM0 [4 mm], VM0 [0.6 mm], ER0) (Analysis by hematoxylin‐eosin staining of representative section 5 Figure 3a–d) (Immunohistochemically, epithelial membrane antigen staining (Figure 4a), Desmin staining showing pT1b [SM, 6000 µm] (Figure 4b). In addition, the tumor cells were positive for D2‐40 staining (Figure 4c) and for CD34 staining (Figure 4d). These immunohistochemical findings suggested IMPC of the colon.

1 month after ESD, additional surgery confirmed the absence of residual tumor or lymph‐node metastasis. In that study, having IPMC components > 10% was associated with a higher proportion of lymph‐node metastasis in the surgical specimens [3]. However, no residual tumor or recurrence was observed in our case, and the patient has remained under careful follow‐up for 3 years to date. Genetic profiling of ESD specimens revealed human epidermal growth factor receptor 2 (HER2) positivity and absence of biomarkers of mutations, RAS, BRAF, and MMR.

## 3 Discussion

IMPC was first described by Siriaunkgul et al. [1] in 1993 as a subtype of invasive ductal carcinoma with high propensity for lymphovascular invasion and lymph node metastasis. Reports of IMPC in organs such as the urinary tract, lungs, and salivary glands have since emerged, and all of them are associated with poor prognosis due to frequent lymphatic spread [2].

A PubMed search for “colorectal” and “micropapillary carcinoma” yielded 43 reported cases as of 2024. Of these, only six cases, including ours, involved T1‐stage colorectal cancer (Table S1) [4, 5, 6, 7, 8, 9]. Despite the intermediate tumor size, significant lymphatic and venous invasion was observed. All patients eventually required surgical resection, and some experienced recurrence or metastasis.

Haupt et al. [3] reported a potential, albeit not statistically significant, correlation between IMPC components, constituting > 10% of the tumor, and prognosis. Similarly, Xu et al. [10] reported that colorectal carcinomas with IMPC components, even at early T1 or T2, frequently exhibit lymphovascular invasion.

This case represents the first reported instance of identification of a cancerous IMPC component by magnifying endoscopy at the T1b (SM) depth. Thus, even for intermediate lesions, IMPC should be considered as a differential diagnosis when endoscopic imaging suggests malignancy.

In the present case, additional surgery revealed no residual tumor or lymph node metastasis. However, clinical questions remain regarding the extent of lymph node dissection and the role of adjuvant chemotherapy in T1b cases after endoscopic resection.

Given that HER2‐positive colorectal cancer, such as in this case, a rare subtype, accounts for 2%–3% of colorectal cancers, and is associated with a poor response to anti‐epidermal growth factor receptor antibody therapy, targeted treatment options must be considered [10]. This underscores the importance of accumulating data on IMPC cases, treatment regimens, and long‐term outcomes to clarify the significance of lymph node dissection and adjuvant therapy in such cases. At present, information on Her2 expression in colon cancer with IMPC is limited, and no clear association has been demonstrated. However, given the findings in IMPC of other organs and the significance of Her2 expression in colon cancer as a whole, future studies are expected to clarify the role of Her2 in colon cancer with IMPC. If Her2 expression is confirmed, the applicability of HER2‐targeted therapy (e.g., trastuzumab) used in breast and gastric cancer may also be considered. Future studies are expected to clarify the frequency and clinical significance of Her2 expression in colon cancer with IMPC.

## Conflicts of Interest

Thr authors declare no conflicts of interest.

## Ethics Statement

Approval of the research protocol by an Institutional Review Board: N/A. Ethical review is not required for case reports of 10 or fewer cases that are retrospective analyses of approved treatments.

## Consent

The patient has consented to the release of this information.

## Clinical Trial Registration

N/A.

## Supporting information




**TABLE S1** Six colonic IMPC cases, including ours, involved T1‐stage colorectal cancer.
